# Analysis of the Distribution Pattern of Remaining Oil and Development Potential after Weak Gel Flooding in the Offshore LD Oilfield

**DOI:** 10.3390/gels10040236

**Published:** 2024-03-29

**Authors:** Lizhen Ge, Xiaoming Chen, Gang Wang, Guohao Zhang, Jinyi Li, Yang Liu, Lixiao Xiao, Yuchen Wen, Weifeng Yuan, Ming Qu, Mingxing Bai

**Affiliations:** 1 Tianjin Branch of CNOOC Ltd., Tianjin 300459, China; 2 Research Institute of Unconventional Petroleum Science and Technology, China University of Petroleum (Beijing), Beijing 102249, China; 3 SANYA Offshore Oil and Gas Research Institute, Northeast Petroleum University, Sanya 572025, China; m.qu@foxmail.com; 4 College of Petroleum Engineering, Northeast Petroleum University, Daqing 163318, China

**Keywords:** weak gel flooding, online nuclear magnetic resonance, microscopic oil displacement, remaining oil, development potential

## Abstract

The LD oilfield is one of the representative offshore oilfields. After weak gel flooding, the recovery rate is significantly improved. However, the oilfield is then in a medium- to high-water content stage, presenting a complex distribution of the remaining oil. The measures for further enhanced oil recovery (EOR) are uncertain. As a result, it is necessary to clarify the distribution pattern and development potential of the remaining oil during the high-water content period after weak gel flooding. In this study, an online nuclear magnetic resonance (NMR) oil displacement experiment and microscopic oil displacement experiment were conducted, and the mechanisms of weak gel flooding and the distribution pattern of the remaining oil were clarified in the LD oilfield. Additionally, high-multiple water flooding and numerical simulation experiments were conducted to analyze the development potential after weak gel flooding. The results show that the effect of weak gel flooding was more significant in the core of 1500 mD, with an increase in oil recovery of 9% compared to 500 mD. At a permeability of 500 mD, the degree of crude oil mobilization in micropores and small pores caused by weak gel flooding was improved by 29.64% and 23.48%, respectively, compared with water flooding. At 1500 mD, the degree of crude oil mobilization in small pores caused by weak gel flooding was increased by 37.79% compared to water flooding. After weak gel flooding, the remaining oil was primarily distributed in medium and large pores. Microscopically, the remaining oil was dominated by cluster residual oil, accounting for 16.49%, followed by columnar, membranous, and blind-end residual oil. High multiple water flooding experiments demonstrated that weak gel flooding could significantly reduce development time. The ultimate oil recovery efficiency of 500 mD and 1500 mD reached 71.85% and 80.69%, respectively. Numerical simulation results show that the ultimate oil recovery efficiency increased from 62.04% to 71.3% after weak gel flooding. This indicated that the LD oilfield still had certain development potential after weak gel flooding. The subsequent direction for enhanced oil recovery focuses mainly on mobilizing oil in medium pores or clustered remaining oil. This will play a crucial role in further exploring methods for utilizing the remaining oil and increasing the recovery rate.

## 1. Introduction

Prolonged water injection in reservoirs often results in the formation of dominant channels, exacerbating reservoir heterogeneity and leading to fluid channeling. This phenomenon reduces the efficiency of waterflooding within the reservoir [1,2,3]. Consequently, deep profile control and displacement in the reservoir are essential to mitigate water channeling and improve reservoir heterogeneity [4,5,6,7].

In recent years, research on reservoir profile control and displacement technologies has primarily focused on polyacrylamide-based plugging and surfactant-based washing [8]. The introduction of polymers or cross-linked polymer gel plugs effectively seals off water channeling pathways and facilitates subsequent water injection to bypass these channels. The addition of surfactants to the injected water reduces the interfacial tension (IFT) or forms emulsions, thereby leading to enhanced oil recovery (EOR). Microemulsions exhibit interesting physicochemical properties and have a wide range of applications [9,10,11]. Moreover, surfactant-based microemulsion flooding, a miscible displacement process, significantly reduces IFT and decreases the capillary forces acting on oil droplets, thereby becoming a potential candidate for EOR [12]. The injection of polymer solutions, despite improving the mobility ratio and expanding the sweep efficiency, faces significant shear forces within porous media, leading to considerable viscosity reduction [8,13]. Core flooding experiments have shown that polymer solutions have a limited ability to alter fluid flow due to shear effects. In summary, the direct application of polymer flooding, surfactant flooding, or microemulsion flooding presents certain limitations in strongly heterogeneous reservoirs, including the risk of breakthrough.

To effectively plug water flow channels, it is common to inject plugging agents with greater sealing strength into water injection wells, thereby improving water injection efficiency and increasing oil recovery. Liu et al. [14] proposed a sodium silicate water shut-off agent that can precipitate in the formation, partially or completely blocking the pores in the formation. Bai et al. [15] proposed a polymer gel plugging agent for in situ cross-linking of HPAM with chromium (Cr^3+^). Zhu et al. [16] recently developed preformed particle gels (PPGs) in which the conformance control mechanism mainly involves physical plugging, including trapping and flocculation. Gallo et al. [17] proposed a resin-type water shut-off agent that can enter the formation after dilution. Under formation temperature, it exhibits extremely high strength after aging and is capable of plugging pores and throats in the formation with the assistance of hardening agents. Recent foreign applications of gel systems, such as in-depth fluid diversion and colloidal dispersion gel techniques, have revolutionized conventional polymer flooding. It can take the advantages of cross-linked polymers to modulate internal flow dynamics in reservoirs [18,19,20,21].

Weak gel-based displacement agents are developed from colloidal dispersion gel and bulk gel systems. This is a three-dimensional network structure consisting of low-concentration polymers, cross-linkers, and stabilizers, which are formed mainly via intermolecular cross-linking, supplemented by intramolecular cross-linking [22,23]. The weak gel systems exhibit excellent injectability, transportability, and deep profile control capabilities, allowing them to plug deep reservoir layers and alter the flow channels of injected water [24,25]. When polymers and cross-linkers are injected into heterogeneous porous media, they selectively flow towards higher permeability layers, forming in situ weak gels, increasing flow resistance, and even blocking high-permeability channels. As a result, the injection pressure gradient is increased, forcing subsequently injected water to overcome capillary forces and enter smaller pore channels, mobilizing the remaining oil [26]. Research by Seright and others [27] has extensively investigated the sealing effect of weak gels on fractures, indicating that partially cross-linked weak gels not only reduce damage to non-target layers but also enhance the sealing effect on fractures. Cheng et al. [28] carried on microscopic visualization experiments and numerical simulations and indicated that weak gels could effectively reduce the water-to-oil mobility ratio. High-permeability layers and large pore channels are selectively blocked, subsequent water flow is forced to redirect, and viscoelastic movement is used to generate negative pressure for oil suction. Cao et al. [29] developed a formula for weak gel systems based on the characteristics of high temperature, high permeability, and large pores in the Y1 block of the Daqing Yushulin Oilfield. These systems undergo cross-linking reactions upon entering deep strata, achieving deep control over oil displacement. Core plugging and oil recovery tests indicated that the gel system could be easily injected into porous media and then formed into gel in situ. Afterwards, the high-permeability layers were selectively plugged, and subsequent water flowed into low-permeability layers, thus improving sweep efficiency and ultimate oil recovery [30]. Wang et al. [31] evaluated weak gel under different conditions, demonstrating its superior profile control and oil displacement performance compared to polymers. Successful field tests of weak gel flooding were conducted in the LD oilfield, with most production wells around the weak gel injection wells responding post-injection. The injection pressure significantly increased, and the average daily oil production of the responding wells increased by 33.2 m^3^/d, indicating a notable increment in oil recovery.

Laboratory experiments and field pilot tests suggest that weak gel deep profile control is a crucial method for improving the sweep efficiency of injected water and enhancing the ultimate recovery of high-water content reservoirs. While extensive research has been conducted on evaluating the effectiveness and mechanisms of weak gel flooding, the distribution patterns of the remaining oil after weak gel flooding and its development potential have not been sufficiently addressed.

At the same time, as oil fields on land or at sea re-enter a period of high-water content following chemical EOR processes, the direction for further EOR techniques remains uncertain. Hence, after chemical flooding such as weak gel or polymer, the research direction of further improving oil recovery has gradually attracted people’s attention. What is more, the distribution of the remaining oil becomes more complex after chemical EOR, posing challenges to the implementation of additional recovery methods. Therefore, to explore directions for further EOR techniques, it is essential to first understand the distribution patterns and development potential of the remaining oil after weak gel flooding. Only by comprehending these distribution patterns can we accurately investigate how to mobilize the remaining oil.

The LD oilfield is located in the Liaodong Bay of the Bohai Sea, in the middle and southern part of the Liaoxi Low Bulge. It belongs to the fractured semi-dorsal tectonic structure developed on the submerged mountains. The provenance is from a northwesterly direction. The depositional environment is the front edge of the Braid River delta, with a predominantly submerged diversion channel developing. The reservoir type is a stratified tectonic reservoir influenced by lithology, with a large reservoir thickness and good physical properties. It is a highly porous, medium-high permeability sandstone reservoir. The reservoir porosity is mainly distributed between 25% and 31%. The permeability is mainly distributed between 400 mD and 2500 mD. As a typical offshore field, it has an exploitation history of twenty years. The development history of LD oilfield showed that when the water cut was 9%, weak gel flooding was carried out to slow down the water cut rise rate. However, it is now entering a period of high-water content. The unclear understanding of distribution patterns of the remaining oil post-weak gel flooding leads to uncertainties regarding further EOR methods post-weak gel flooding.

In addition, according to the usual experimental specifications, the experiment of water flooding generally ends after the water cut reaches 99.95% or displaces 30 PV. However, for offshore reservoirs of high porosity and high permeability, the displacement efficiency could be greatly enhanced through high-intensity and high-multiplicity (far beyond 30 PV) water flooding processes. Meanwhile, there is an increasing trend in the core recovery degree as displacement multiplies. In this case, accurately assessing the development potential of offshore reservoirs is difficult based on general experimental specifications. Li et al. [32] pointed out that for heavy oil reservoirs with bottom water in Bohai Oilfield, especially in the high-water content stage, water flooding with a high multiplicity of 2000 PV can significantly improve the oil displacement efficiency. Therefore, the development potential of the Bohai LD oilfield can be more comprehensively analyzed after weak gel flooding via limited water flooding (2000 PV high-multiple water flooding).

In response to the challenges faced by the LD oilfield during the high-water content period following weak gel flooding (such as the unclear distribution patterns of remaining oil), the current potential for further development, and the ambiguous direction for further EOR techniques, this study employed experimental simulations to trace the developmental history: at a water cut of 9%, a 0.4 PV of weak gel was injected.

Firstly, this study employed nuclear magnetic resonance online displacement experiments and microscopic displacement experiments to simulate the weak gel flooding process in the offshore LD oilfield. It aimed to determine the effects and mechanisms of weak gel flooding under different permeabilities and understand the microscopic distribution patterns of the remaining oil post-weak gel flooding. Furthermore, the development potential was analyzed through 2000 PV high-multiple water flooding and numerical simulations in the offshore LD oilfield. By clarifying the distribution patterns of the remaining oil post-weak gel flooding and the development potential in the offshore LD oilfield, it lay on a significant foundation for further EOR techniques in offshore oil fields following weak gel flooding.

## 2. Results and Discussion

### 2.1. Evaluation of Weak Gel Flooding Effect

#### 2.1.1. Weak Gel Displacement NMR T_2_ Spectrum

**Figure 1:**
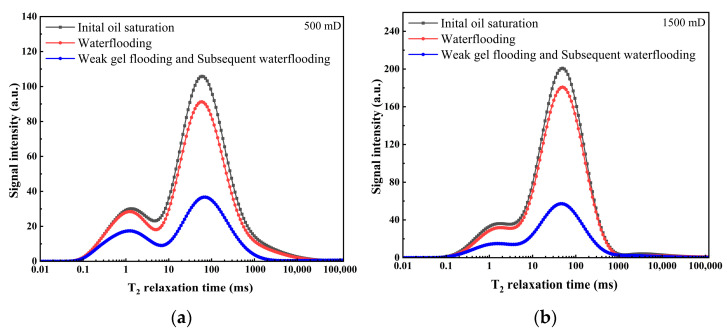
T_2_ spectrum of weak gel flooding: (**a**) 500 mD core; (**b**) 1500 mD core.

This study selected representative natural core samples from the LD oilfield, with permeabilities of 500 and 1500 mD, extracted from a depth of 1581 m, to simulate the development process of weak gel profile control. When the water cut reached 9% during the initial water flooding, 0.4 PV of weak gel was injected, followed by subsequent water flooding. The results of the weak gel flooding NMR online displacement experiment are depicted in Figure 1.

From Figure 1a, it is evident that at a permeability of 500 mD, there is a significant reduction in the T_2_ spectrum curve after water flooding of 0.4 PV, mainly around a relaxation time of 100 ms, indicating a relatively low degree of water drive. The oil displacement efficiency of water flooding is 13.8%. After injecting 0.4 PV of weak gel, the overall T_2_ spectrum curve shows a marked decline, with the oil displacement efficiency during the weak gel flooding stage increasing to 60.62%, an improvement of 46.82%. This efficiency boost is attributed to the selective nature of the weak gel, which preferentially enters medium to large pores, increasing the viscosity of the displacing phase and exhibiting viscoelastic properties, effectively displacing the crude oil from medium and large pores. The significant decrease in signal quantity for relaxation times of 10~100 ms and 100~1000 ms supports this observation.

Furthermore, the reduction in signal quantity for relaxation times of 1~10 ms is mainly due to the weak gel increasing the flow resistance in high-permeability channels, forcing the injected fluid to flow towards low-permeability channels, thus enhancing oil displacement efficiency in smaller pores. During the subsequent water flooding stage, the signal quantity for relaxation times of 1~10 ms further decreases as the weak gel blocks high-permeability channels, forcing subsequent injected water towards lower-permeability channels, thereby further improving oil displacement efficiency in smaller pores.

For the core with a permeability of 1500 mD, the oil displacement efficiency during waterflooding was 10.6%. After injecting 0.4 PV of weak gel, the displacement efficiency increased to 68.8%, an improvement of 58.2%. The trend of the T_2_ spectrum curve during different stages was essentially consistent with that observed in the 500 mD core. Due to the more severe heterogeneity in the high-permeability core, the effect of weak gel profile control was more pronounced, with the oil displacement efficiency during the weak gel flooding stage being 9% higher compared to the 500 mD core. Consequently, the weak gel has a significant profile control effect in both 500 mD and 1500 mD cores, effectively controlling the channeling pathways and improving oil displacement efficiency.

#### 2.1.2. Utilization of Crude Oil in Pores of Different Sizes

In the nuclear magnetic resonance (NMR) T_2_ spectrum, the horizontal axis representing relaxation time corresponds to the sizes of pores within the core, while the vertical axis signal amplitude indicates the oil content in pores of different sizes. The relaxation times of 0.1~1, 1~10, 10~100, and 100~1000 ms are defined as micropores, small pores, medium pores, and large pores, respectively. By analyzing the signal quantity in pores of various sizes, the effect of weak gel on mobilizing crude oil in different pore sizes can be assessed.

**Figure 2:**
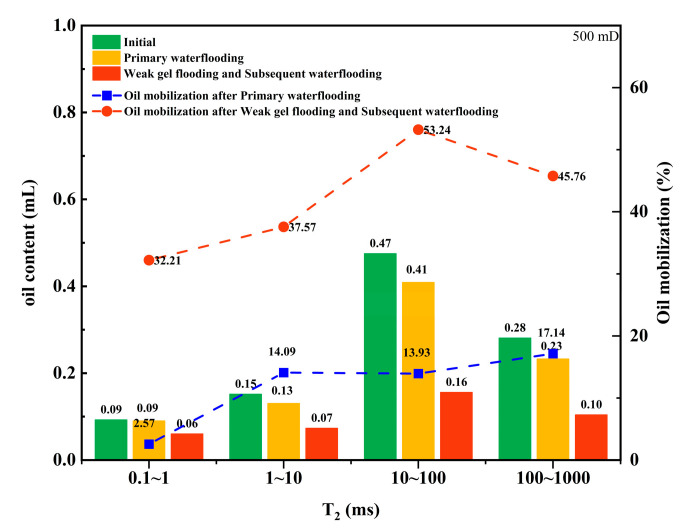
Oil content and mobilization degree of various pores in different stages (500 mD).

**Figure 3:**
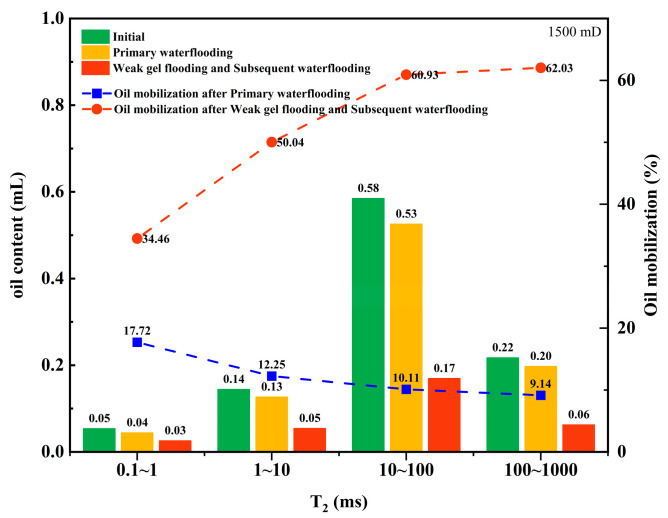
Oil content and mobilization degree of various pores in different stages (1500 mD).

Figure 2 and Figure 3 illustrate the mobilization of crude oil in different pore sizes during water flooding and weak gel flooding stages in cores with different permeabilities. From Figure 2, for a core with a permeability of 500 mD, the overall degree of mobilization during the water flooding stage is relatively low, with micropores, small pores, medium pores, and large pores showing mobilization of 2.57%, 14.09%, 13.93%, and 17.14%, respectively. During the weak gel flooding stage, there is noticeable mobilization of oil in micropores and small pores, increasing to 32.21% and 37.57%, respectively. The degree of oil mobilization in medium and large pores is higher, at 53.24% and 45.76%, respectively. This indicates that the weak gel preferentially enters high-permeability channels, fully mobilizing oil in medium and large pores. Subsequently, by increasing the flow resistance in high-permeability channels, the fluid is redirected towards lower-permeability channels, enhancing the mobilization of oil in medium and small pores. For a core with a permeability of 1500 mD, as shown in Figure 3, the degrees of mobilization during the water flooding stage for micropores, small pores, medium pores, and large pores are 17.72%, 12.25%, 10.11%, and 9.14%, respectively. During the weak gel flooding stage, the degrees of mobilization in micropores, small pores, medium pores, and large pores increase to 34.46%, 50.04%, 60.93%, and 62.03%, respectively.

**Figure 4:**
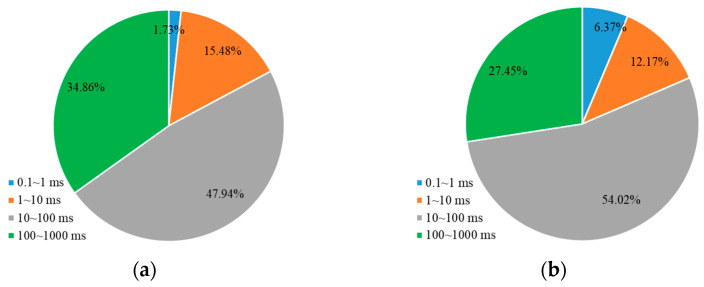
The percentage of oil production from different pore sizes relative to the total oil production (500 mD): (**a**) waterflooding; (**b**) weak gel and subsequent waterflooding.

Furthermore, the contribution of oil production from different pore sizes during the water flooding and weak gel flooding stages was analyzed. This refers to the proportion of oil produced from each pore relative to the total oil produced. As shown in Figure 4a, for a core with a permeability of 500 mD during the water flooding stage, the majority of the produced oil comes from medium and large pores, accounting for 47.94% and 34.86%, respectively, followed by small pores at 15.48%. The contribution from micropores is the lowest at only 1.73%. After the injection of weak gel, as depicted in Figure 4b, during the weak gel flooding stage for a 500 mD core, due to the higher initial oil content in medium and large pores, the majority of produced oil still comes from these pores, with contribution rates of 54.02% and 27.45%, respectively. The contribution from small pores increased to 12.17%, and the contribution from micropores increased from 1.71% to 6.37%. This indicates that the weak gel effectively blocked high-permeability channels, forcing the fluid to flow towards lower-permeability channels, thereby enhancing the oil displacement efficiency in smaller pores.

**Figure 5:**
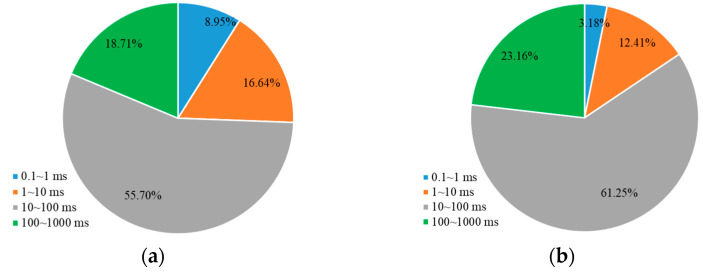
The percentage of oil production from different pore sizes relative to the total oil production (1500 mD): (**a**) waterflooding; (**b**) weak gel and subsequent waterflooding.

For the core with a permeability of 1500 mD, as shown in Figure 5, during the water flooding stage, the majority of the produced oil comes from medium pores, accounting for 55.70% of the total. The contributions from small and large pores are relatively close at 16.64% and 18.71%, respectively, with micropores contributing 8.95%. During the weak gel flooding stage, as indicated in Figure 5b, the contribution from medium pores increases to 61.25%, while large pores contribute 23.16%. The contributions from small pores and micropores are 12.41% and 3.18%, respectively. This suggests that in a higher-permeability core, medium and large pores play a more significant role in oil production, and the use of weak gel enhances the mobilization and production of oil from these pores.

### 2.2. Mechanism of Weak Gel Flooding

#### 2.2.1. Profile Control

Initially, due to the viscosity properties of the weak gel, the capillary number was increased during the weak gel injection phase, further mobilizing the remaining oil in high-permeability channels, with the mobilization degree of remaining oil in large pores increasing from 17.14% to 45.76% (Figure 2). Theoretically, the capillary number during weak gel flooding should only increase by a few times compared to water flooding and is effective only during the weak gel injection phase, which would not significantly enhance oil displacement efficiency. However, the experimental results (Figure 2 and Figure 3) show a significant improvement in displacement efficiency across cores with varying permeabilities. Thus, a more crucial mechanism by which weak gel flooding significantly enhances recovery is through its deep profile modification capabilities. The weak gel preferentially enters high-permeability channels with lower flow resistance, further mobilizing crude oil in medium and large pores. Additionally, due to the higher viscosity of weak gel compared to water, the flow resistance in high-permeability channels is increased, potentially even blocking these channels, leading to an increased injection pressure gradient. This forces the subsequently injected water to overcome the capillary forces in small pores, thereby mobilizing the remaining oil in micropores and small pores of the core. In the 500 mD core, the mobilization degree of the remaining oil in micropores and small pores increased from 2.57% to 32.21% and from 14.09% to 37.57%, respectively (Figure 2). In the 1500 mD core, the mobilization degree of the remaining oil in micropores and small pores increased from 17.72% to 34.46% and from 12.25% to 50.04%, respectively (Figure 3).

#### 2.2.2. Microscopic Mechanism

**Figure 6:**
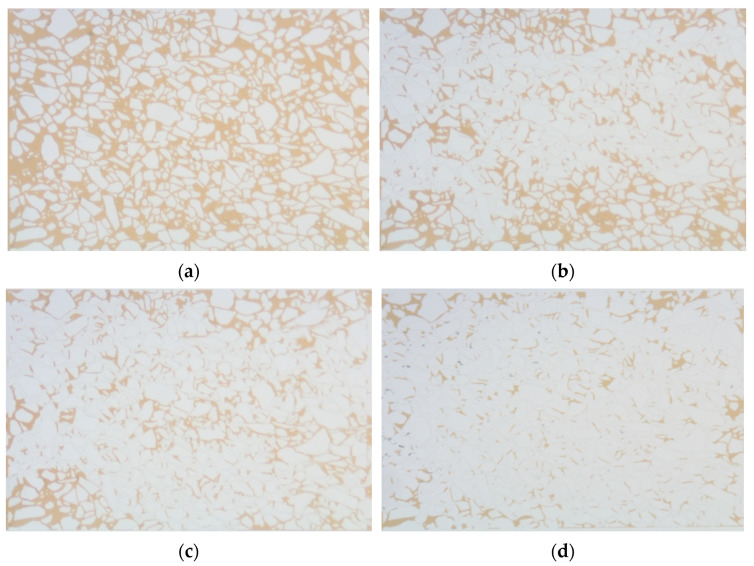
Weak gel micro-displacement process: (**a**) initial state; (**b**) water flooding forms a dominant channel; (**c**) weak gel flooding; (**d**) subsequent water flooding.

**Figure 7:**
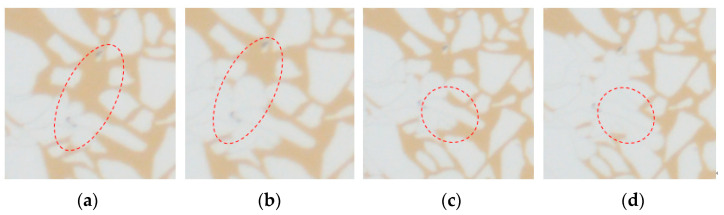
Microscopic flow process of weak gel: (**a**,**b**) weak gel selects into large pore; (**c**,**d**) the flow is diverted.

Based on the thin cast sections of natural cores from the LD oilfield, a microscopic visualization model was created to conduct weak gel microscopic oil displacement experimental research. As indicated in Figure 6, during the initial water flooding, the injected water first flows along high-permeability channels, forming dominant channels in the central part of the model. After the injection of weak gel, the remaining oil in the peripheral regions of the model begins to be mobilized. In the subsequent water flooding stage, the remaining oil in these peripheral areas is further effectively mobilized. A detailed analysis of the microscopic model, particularly after the injection of weak gel, reveals the following, as shown in Figure 7a,b: the weak gel initially prefers to enter larger pores, thereby increasing the flow resistance in dominant channels. This alteration forces the subsequent fluid to flow towards areas that have not yet been reached by the initial water flooding (as depicted in Figure 7c,d), initiating the mobilization of remaining oil in the peripheral regions of the model. This process illustrates the effectiveness of weak gel in altering the flow paths within the reservoir and enhancing oil recovery.

### 2.3. Distribution Patterns of Remaining Oil after Weak Gel Flooding

**Figure 8:**
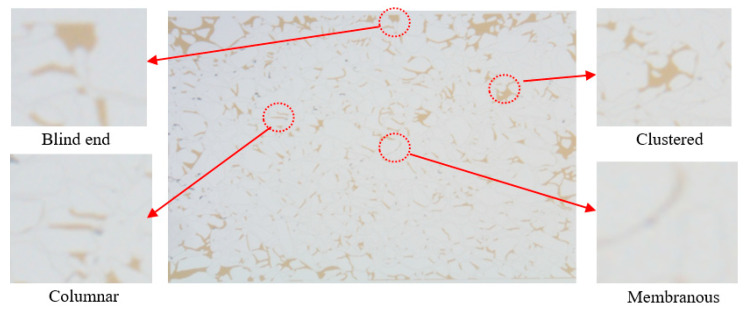
Types of microscopic remaining oil after weak gel flooding. (The red circles represent different types of remaining oil).

The online nuclear magnetic resonance (NMR) oil displacement and microscopic oil displacement experiments demonstrate that weak gel profile control is significantly effective. However, after weak gel profile control, some of the remaining oil remains undisturbed in the core. Understanding the distribution pattern of this remaining oil after weak gel profile control is crucial to guiding further research and application in oil field development. An analysis of the remaining oil post-weak gel flooding, as shown in Figure 8, reveals that the distribution of remaining oil is scattered, with types including clustered, columnar, membranous, and dead-end remaining oil. A statistical analysis, following the identification of different types of remaining oil, indicates that clustered remaining oil is the predominant form in the core’s pore channels post-weak gel profile control, accounting for 16.49%. This is followed by membranous remaining oil at 9.55%, columnar remaining oil at 5.89%, and dead-end remaining oil, constituting only 0.72%. The distribution pattern of microscopic remaining oil suggests that the primary target for mobilization post-weak gel profile control should be clustered remaining oil.

**Figure 9:**
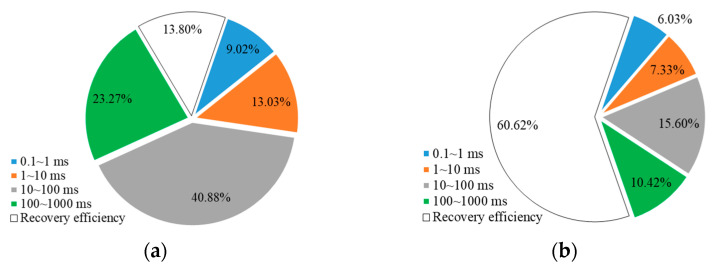
Distribution of remaining oil in pores at different stages and stage recovery efficiency (500 mD): (**a**) waterflooding; (**b**) weak gel and subsequent waterflooding.

**Figure 10:**
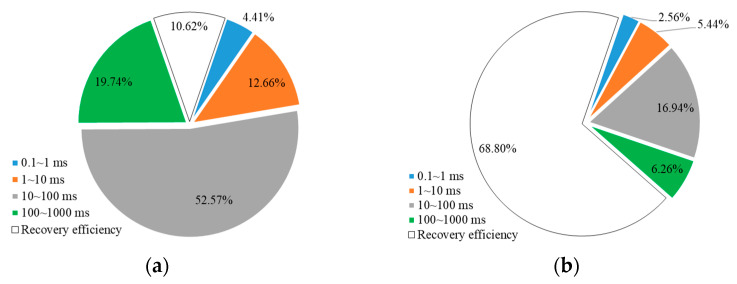
Distribution of remaining oil in pores at different stages and stage recovery efficiency (1500 mD): (**a**) waterflooding; (**b**) weak gel and subsequent waterflooding.

Further analysis was conducted on the remaining oil content in different pore sizes of cores with varying permeabilities after weak gel profile control. This analysis was similar to the method described in Section 2.1.2, as depicted in Figure 9 and Figure 10. For a core with a permeability of 500 mD, during the water flooding stage, the produced crude oil accounted for 13.80% of the initial saturated oil. The remaining oil in micropores, small pores, medium pores, and large pores constituted 9.02%, 13.03%, 23.27%, and 40.88% of the initial saturated oil, respectively. During the weak gel profile control stage, the cumulative produced crude oil accounted for 60.62%, with the remaining oil in micropores, small pores, medium pores, and large pores constituting 6.03%, 7.33%, 15.60%, and 10.42%, respectively. For a core with a permeability of 1500 mD, during the water flooding stage, the produced crude oil accounted for 10.62% of the initial saturated oil. The remaining oil in micropores, small pores, medium pores, and large pores constituted 4.41%, 12.66%, 52.57%, and 19.74% of the initial saturated oil, respectively. During the weak gel profile control stage, the cumulative produced crude oil accounted for 68.80%, with the remaining oil in micropores, small pores, medium pores, and large pores constituting 2.56%, 5.44%, 16.94%, and 6.26%, respectively.

After weak gel profile control, the remaining oil is still primarily distributed in medium and large pores. This is because the weak gel’s displacement distance is limited, and its profile control action is mainly concentrated in the front half of the core, resulting in a limited effect in the latter half and leaving a substantial amount of remaining oil. Additionally, the proportion of remaining oil in micropores and small pores increases. For a core with a permeability of 500 mD, during the water flooding stage, the remaining oil in micropores and small pores accounted for 9.02% and 13.03% of the total remaining oil, respectively. At the end of the weak gel profile control stage, these percentages increased to 15.34% and 18.61%. For a core with a permeability of 1500 mD, the proportion of remaining oil in micropores increased from 4.93% to 8.21%, and the proportion in small pores increased from 14.16% to 17.44%. Therefore, after weak gel profile control, the primary targets for further enhanced oil recovery efforts are medium and large pores. However, attention should also be given to the remaining oil in micropores and small pores, indicating a need for more refined strategies to address these challenging areas for improved oil recovery.

### 2.4. Analysis of Development Potential after Weak Gel Flooding in Offshore LD Oilfield

After implementing weak gel profile control in the Bohai LD oilfield, significant increases in injection pressure and enhanced oil recovery were observed. However, the field has now entered a mid-to-high water cut stage, with a water cut of 87.4% as of February 2023. It is imperative to analyze the development potential of the LD oilfield. By further analyzing the ultimate oil displacement efficiency and target recovery rates post-weak gel profile control, the long-term development effects can be predicted. Understanding the development potential post-weak gel profile control in the Bohai LD oilfield, combined with the distribution patterns of remaining oil after such control, is crucial for guiding further research on enhancing recovery rates.

#### 2.4.1. Evaluation of the Ultimate Oil Displacement Efficiency

**Figure 11:**
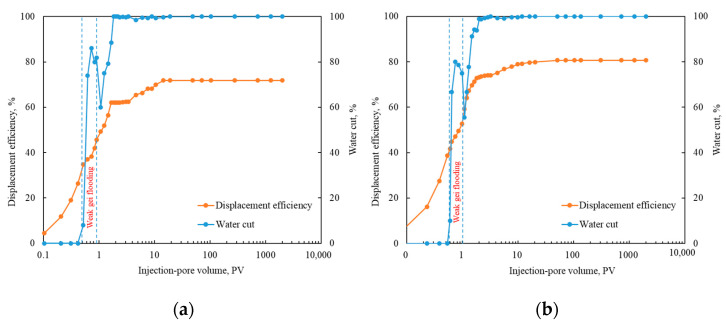
A high-multiple water flooding experiment of 2000 PV: (**a**) 500 mD core; (**b**) 1500 mD core.

Simulating the weak gel flooding process of the LD oilfield, 0.4 PV of weak gel was injected when the water cut reached 9%, followed by a high multiple water flooding experiment of 2000 PV. The change in oil displacement efficiency with the injected PV number was recorded, with results shown in Figure 11. After the injection of weak gel, the oil displacement efficiency rapidly increased. After 2000 PV of high multiple water flooding, the ultimate oil displacement efficiencies for 500 mD and 1500 mD were 71.85% and 80.69%, respectively. The ultimate oil displacement efficiency for single water flooding ranged between 68.8% and 76.5% [23]. After weak gel flooding, the increase in ultimate oil displacement efficiency was not significant. However, the corresponding PV number for reaching a near-flat efficiency curve significantly reduced from 100 PV in water flooding to 14~20 PV post-weak gel flooding [23]. Given the limited operational life of offshore platforms, weak gel flooding can substantially reduce development time costs.

Furthermore, the current water cut of the Bohai LD Oilfield is 87.4%, with oil displacement efficiencies for 500 mD and 1500 mD being 60.86% and 69.12%, respectively. When the water cut reaches 98%, the displacement efficiencies could increase to 62% and 73.12%, respectively. In the ultimate water flooding scenario, these efficiencies could further rise to 71.85% and 80.69%. Considering the current development status of the LD Oilfield, the oil displacement efficiency could potentially be improved by approximately 11%, indicating that the Bohai LD Oilfield still has significant development potential post-weak gel profile control.

#### 2.4.2. Evaluation of Target Recovery Rate

**Figure 12:**
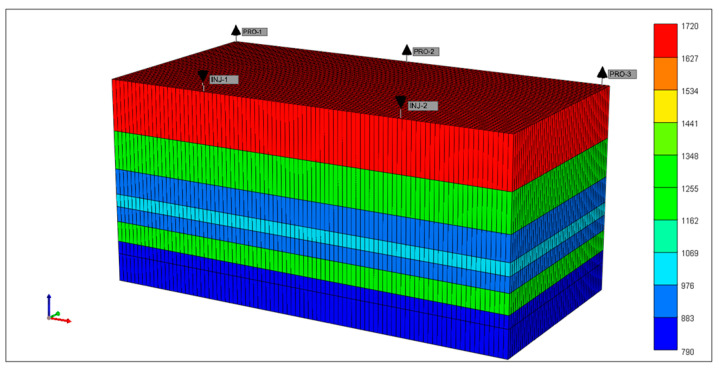
Establishment of the conceptual model.

An in-depth analysis of the LD oilfield’s development potential post-weak gel flooding was conducted using CMG numerical simulation methodology. This analysis focused on crucial indicators such as the target recovery rate and maximum sweep coefficient. Leveraging geological, lithological, and reservoir properties, as well as development methods data specific to the LD oilfield (refer to Table 1), a conceptual pattern of a row–column staggered well network model was established. The foundational geological parameters comprised a five-spot well pattern, well spacing of 200 m, an 80 m reservoir thickness, and vertical division into eight sub-layers (from top to bottom: 20 m, permeability of 1720 mD; 15 m, 1320 mD; 10 m, 970 mD; 5 m, 1040 mD; 6 m, 970 mD; 8 m, 1280 mD; 5 m, 850 mD; 11 m, 790 mD). The model’s total volume was 6200 × 10^4^ m^3^, as illustrated in Figure 12. This model serves as a basis for simulating the weak gel profile control process.

**Figure 13:**
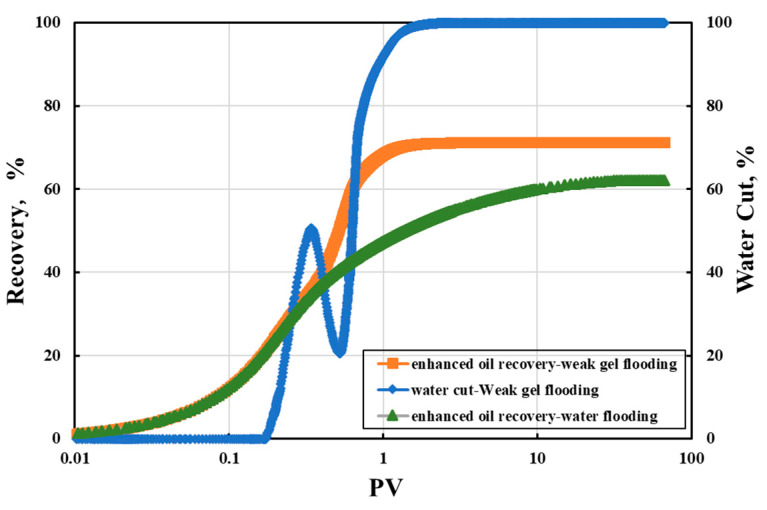
Recovery efficiency and water cut under water flooding and weak gel flooding.

Simulations of water flooding and weak gel flooding were conducted to determine the target recovery rates and maximum sweep efficiencies under different development methods, thereby assessing the development potential of the LD Oilfield. The results of the simulations for water flooding and weak gel flooding are depicted in Figure 13.

During the initial phase of water flooding, the recovery rate increases slowly, followed by a rapid increase. The recovery rate reaches 11.05% when the cumulative injection volume is 0.09 PV. It continues to increase swiftly, reaching 38.9% at a cumulative injection of 0.45 PV. Subsequently, the rate of increase slows down, and the target recovery rate ultimately reaches 62.04% when the cumulative injection volume is 31.41 PV. The long-term water flooding simulation of the LD Oilfield indicates that the increase in recovery rate slows down in the later stages of development. Additionally, after the limit of water flooding is reached, a significant amount of remaining oil remains unexploited. Given the constraints of the operational lifespans of offshore oilfield platforms, long-term water flooding in the LD Oilfield has certain limitations.

Further assessment of weak gel flooding’s effect on the LD Oilfield was conducted by simulating the reservoir history. Upon reaching a water cut of 9% during water flooding, 0.4 PV of weak gel was injected, followed by continued water flooding. The simulation results are illustrated in Figure 13. It is observable from Figure 13 that after the initial injection of weak gel, the rate of recovery increased gradually but then rapidly escalated, ultimately reaching a maximum recovery rate of 71.3%. This represents a 9.26% increase compared to water flooding. Notably, the water cut rose rapidly to 50.23% upon reaching an accumulated injection volume of 0.33 PV, followed by a decline to 20.93% due to the weak gel blocking the high-permeability channels. During this phase, the rapid increase in the recovery rate was attributed to the weak gel entering the porous media of the reservoir, primarily infiltrating the high-permeability layers. Chemical adsorption and mechanical trapping caused the weak gel to be retained in these high-permeability layers, generating additional flow resistance and thereby expanding the affected volume. As the single-phase water cut reached 95%, employing reservoir engineering techniques computed a strong affected volume of 23.65% and a moderate affected volume of 76.35% during water flooding, indicating limited strong affected areas due to the formation of water dominant channels. In contrast, during weak gel flooding at a 95% water cut, the calculated strong affected volume was 93.69%, with a weak affected volume of 6.31%. Compared to water flooding, weak gel flooding expanded the affected range by 70.04%.

Moreover, the minimum PV required to approach the target recovery rate for weak gel flooding was 1.28 PV, significantly lower than the 15 PV for water flooding, substantially reducing development time. Results from ultimate oil displacement experiments and numerical simulations indicate that compared to pure water flooding, weak gel flooding exhibits better development potential. Post-weak gel flooding, the oil recovery efficiency of the LD oilfield can be enhanced to 71.85% and 80.69%, consequently increasing the target recovery rate from 62.04% to 71.3%.

## 3. Conclusions

Understanding the remaining oil distribution pattern and development potential post-weak gel flooding in the offshore LD oilfield holds crucial significance in guiding further studies aimed at EOR. The effects of weak gel flooding in the LD oilfield are remarkable.

1. Experimental findings indicate that weak gel preferentially enters the intermediate to large pores (10~100 ms, 100~1000 ms), amplifying the flow resistance in high-permeability channels. This compels injected fluids to flow towards low-permeability channels, thereby enhancing the utilization of smaller pores (1~10 ms).
2. Post-weak gel flooding, the remaining oil is predominantly found in the intermediate to large pores while the distribution of microscopic remaining oil is scattered, predominantly consisting of cluster-shaped oil, accounting for 16.49%, followed by columnar, membranous, and dead-end oil.
3. To further increase the oil recovery, the focus should be on exploiting the remaining oil in the intermediate pores or predominantly targeting cluster-shaped and membranous residual oil. In comparison to the current development status of the LD Oilfield, the oil displacement efficiency can be enhanced by approximately 11%.
4. Moreover, the maximum target oil recovery can reach 71.3%, indicating that even post-weak gel flooding, the offshore LD oilfield still retains a certain level of development potential.

## 4. Materials and Methods

To investigate the distribution patterns and development potential of remaining oil during the high water cut period after weak gel flooding in the LD oilfield, this study explores the distribution of remaining oil on both the macroscopic (using real core models) and microscopic scales. Initially, displacement experiments were conducted using natural cores and crude oil to simulate the displacement history of the LD oilfield, ensuring experimental repeatability. The first phase involved water flooding until the water cut at the core outlet reached 9%, marking the transition to the second phase of injecting 0.4 PV of weak gel. The third phase resumed water flooding until the water cut reached 98%, at which point the experiment was terminated. Nuclear magnetic resonance scanning tests were performed at different displacement stages to analyze the T_2_ spectrum curves, where the relaxation time on the *x*-axis represented pores of different sizes in the core and the signal intensity on the *y*-axis indicated oil content, facilitating the characterization of the distribution and changes in remaining oil within different pores.

Furthermore, based on the thin cast sections of the LD oilfield’s natural cores, the pore throat structures were extracted to create microscopic models that represent the core’s real microscopic structure, enhancing the experiment’s repeatability. Subsequent microscopic visual displacement experiments involved water flooding, weak gel flooding, and subsequent water flooding to identify the types and patterns of remaining oil distribution at different stages.

Lastly, to further explore the development potential of the LD oilfield, considering the uniqueness of offshore fields where platforms have limited lifespans, often leading to aggressive injection and production strategies, using a water cut of 98% as the endpoint for displacement experiments may not be suitable for offshore fields. In other words, when the water cut reaches 98%, the water injection volume is generally a few PVs. Typically, the water injection multiples at the end of development in offshore fields far exceed those in the experiments. Thus, to more accurately assess the development potential of the LD oilfield under extreme conditions, this study extended water flooding beyond a 98% water cut until reaching an injection multiple of 2000 PV, thereby determining the ultimate recovery rates under extreme water flooding conditions in the LD oilfield. The detailed experimental steps are as follows.

### 4.1. Experimental Materials and Instruments

**Figure 14:**
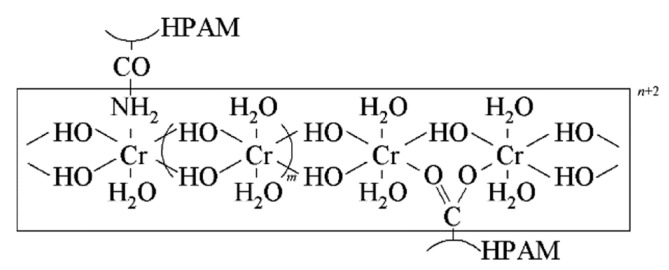
A schematic diagram of the weak gel molecular structure [31].

The experimental oil was simulated to be composed of crude oil and kerosene, and the simulated oil viscosity was 14 cP at 65 °C. The experimental water was simulated formation water with a salinity of 1924 mg/L. The experimental weak gel was composed of a polymer (1200 mg/L) and a chromium cross-linking agent, with a mass ratio of polymer to Cr^3+^ of 180:1. When the shear rate was 7.34 s^−1^, the apparent viscosity of weak gels was 8 mPa·s. For a more detailed description of the rheological properties, refer to Reference [33]. The polymer utilized was partially hydrolyzed polyacrylamide (HPAM) with a molecular weight of 2000 × 10^4^, an effective cost content of 90%, and a hydrolysis degree of 25%, procured from Beijing ZKCHEM Co., LTD, Beijing, China. The cross-linker employed was organic chromium, with an active Cr^3+^ concentration of 3.6%. Firstly, Cr^3+^ formed polynuclear olation complex ions through a complexation reaction, hydrolysis reaction, and olation reaction. Then, polynuclear olation complex ions coordinated with –CONH_2_ and –COO^−^ in HPAM to fabricate a weak gel possessing a reticular structure. For a more detailed description of the reaction process, refer to Reference [31]. A schematic diagram of the weak gel molecular structure is depicted in Figure 14.

The experimental sandstone cores were from the lower part of the East Second Section of the LD oilfield. The termination depth of the core was 1581 m in the lower part of the East Second Section. The average permeabilities were 500 mD and 1500 mD, respectively. The LD oilfield belongs to the fractured semi-dorsal tectonics developed on the submerged mountains. The main oil-containing reservoir in the lower part of the East Second Section is deposited on the front edge of the braided river delta. It is dominated by the development of submerged, diverging river sand bodies.

The experimental equipment includes a microscopic displacement apparatus comprising a microscopic model holder, an intermediate container, a micro displacement pump, and a microscope. Additionally, a nuclear magnetic resonance imaging analyzer (MesoMR23-60H, Newmay Instruments Co., Ltd., Suzhou, China) was used for analysis. The displacement experimental setup involved a temperature-controlled chamber, core holder, ISCO high-precision displacement pump, pressure sensor, and intermediate container, among other components.

### 4.2. Experimental Methods

#### 4.2.1. Online Nuclear Magnetic Resonance (NMR) Oil Displacement Experiment

1. Natural core samples were cleaned, dried, and had their basic parameters measured. The cores were subjected to vacuum treatment, followed by saturation with simulated formation water. After saturation, the T_2_ spectrum of the nuclear magnetic resonance was scanned.
2. The cores were displaced with simulated formation water containing Mn^2+^ ions at a constant rate to thoroughly displace the original water and eliminate the water signal within the cores.
3. Simulated formation water was displaced with simulated oil at a constant rate until the oil content at the outlet of the core reached 100%. This established the initial oil saturation, and the nuclear magnetic resonance T_2_ spectrum was measured to determine the original oil saturation.
4. Water flooding was conducted using simulated formation water containing Mn^2+^ ions at a constant flow rate of 0.2 mL/min, simulating the oil field process. After injecting the weak gel, water flooding was continued until the water saturation reached 98%. The NMR T_2_ spectrum was measured during the displacement process, alongside recording the liquid production and oil recovery at different time intervals.

#### 4.2.2. Microscopic Displacement Experiment

1. Based on the cast thin sections of natural cores from Field LD, pore throat distribution characteristics were extracted to simulate etched microscopic glass models.
2. The model was treated with dimethyldichlorosilane for 48 h at 60 °C, treating the pore structure of the microscopic model to alter its wettability.
3. The model was saturated with simulated oil at a constant rate of 0.02 mL/min. Subsequently, the simulation of the field displacement process began with a constant injection rate of 0.05 mL/min, involving water flooding, weak gel flooding, and subsequent water flooding.

#### 4.2.3. Evaluation of the Ultimate Oil Recovery Efficiency Experiment

Following step 4 of Section 4.2.1, water flooding continued until 2000 PV were displaced while recording the liquid production at the outlet of the core.

## Figures and Tables

**Table 1:** Conceptual model parameters.

Parameters	Value
Grid step size (m)	1550 × 500 × 80
Number of grids	91 × 50 × 8
Porosity (%)	26
Permeability variation coefficient	0.275
Mean permeability (mD)	1117.5
Oil saturation	0.65
Reservoir thickness (m)	80
Crude oil viscosity (mPa·s)	14
Formation water viscosity (mPa·s)	0.46
Weak gel injection (PV)	0.4

## Data Availability

Data are available from the corresponding author upon reasonable request.
